# Supplementary material for: Genomic analysis provides novel insights into diversification and taxonomy of Allorhizobium vitis (i.e. Agrobacterium vitis)
Source: BMC Genomics. 2022 Jun 22;23:462. doi: 10.1186/s12864-022-08662-x (PMC9219206; doi:10.1186/s12864-022-08662-x)
Supplement: Supplementary file 1 — Additional file 1: Fig. S1. Maximum-likelihood core-genome phylogeny of 69 strains belonging to the genus Allorhizobium and other Rhizobiaceae members (uncollapsed). The tree was estimated with IQ-TREE from the concatenated alignment of 344 top-ranked genes selected using GET_PHYLOMARKERS software. The numbers on the nodes indicate the approximate Bayesian posterior probabilities support values (first value) and ultra-fast bootstrap values (second value), as implemented in IQ-TREE. The tree was rooted using the Mesorhizobium spp. sequences as the outgroup. The scale bar represents the number of expected substitutions per site under the best-fitting GTR+F+ASC+R6 model. The same tree, but with collapsed clades, is presented in Figure 1. [file 12864_2022_8662_MOESM1_ESM.pdf]

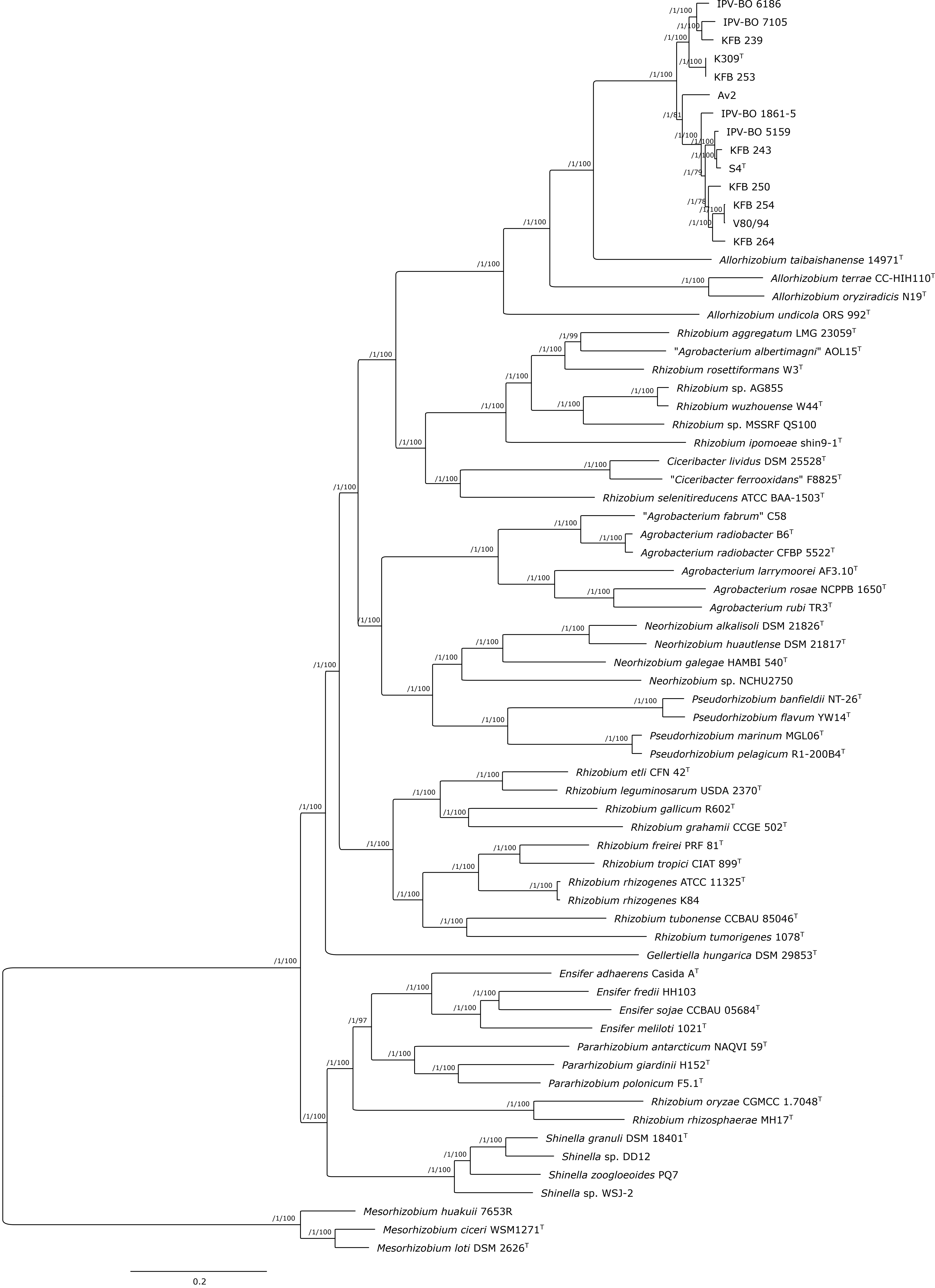

Fig. S1. Maximum-likelihood core-genome phylogeny of 69 strains belonging to the genus *Allorhizobium* and other *Rhizobiaceae* members (uncollapsed). The tree was estimated with IQ-TREE from the concatenated alignment of 344 top-ranked genes selected using GET_PHYLOMARKERS software. The numbers on the nodes indicate the approximate Bayesian posterior probabilities support values (first value) and ultra-fast bootstrap values (second value), as implemented in IQ-TREE. The tree was rooted using the *Mesorhizobium* spp. sequences as the outgroup. The scale bar represents the number of expected substitutions per site under the best-fitting GTR+F+ASC+R6 model. The same tree, but with collapsed clades, is presented in Figure 1.
