# Supplementary figures and images for: Genomic analysis provides novel insights into diversification and taxonomy of Allorhizobium vitis (i.e. Agrobacterium vitis)
Source: BMC Genomics. 2022 Jun 22;23:462. doi: 10.1186/s12864-022-08662-x (PMC9219206; doi:10.1186/s12864-022-08662-x)

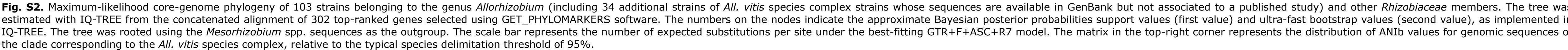

Supplement: Supplementary file 2 — Additional file 2: Fig. S2. Maximum-likelihood core-genome phylogeny of 103 strains belonging to the genus Allorhizobium (including 34 additional strains of All. vitis species complex strains whose sequences are available in GenBank but not associated to a published study) and other Rhizobiaceae members. The tree was estimated with IQ-TREE from the concatenated alignment of 302 top-ranked genes selected using GET_PHYLOMARKERS software. The numbers on the nodes indicate the approximate Bayesian posterior probabilities support values (first value) and ultra-fast bootstrap values (second value), as implemented in IQ-TREE. The tree was rooted using the Mesorhizobium spp. sequences as the outgroup. The scale bar represents the number of expected substitutions per site under the best-fitting GTR+F+ASC+R7 model. The matrix in the top-right corner represents the distribution of ANIb values for genomic sequences of the clade corresponding to the All. vitis species complex, relative to the typical species delimitation threshold of 95%. [file 12864_2022_8662_MOESM2_ESM.pdf]

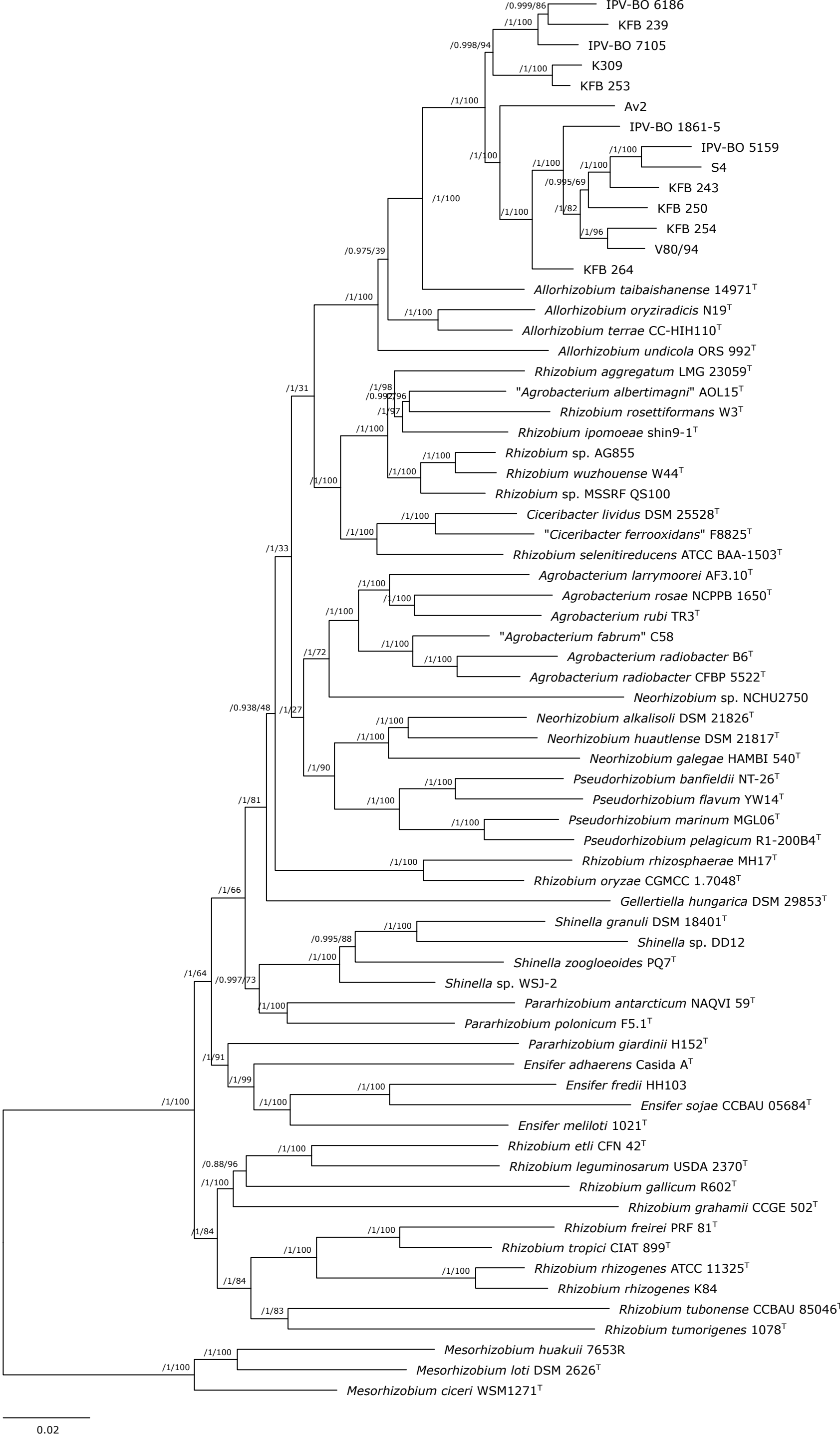

Supplement: Supplementary file 3 — Additional file 3: Fig. S3. Maximum-likelihood pan-genome phylogeny of 69 strains belonging to the genus Allorhizobium and other Rhizobiaceae members (uncollapsed). The tree was estimated with IQ-TREE from the consensus (COGtriangles and OMCL clusters) pan-genome matrix containing 33,396 clusters obtained using GET_HOMOLOGUES software. The numbers on the nodes indicate the approximate Bayesian posterior probabilities support values (first value) and ultra-fast bootstrap values (second value), as implemented in IQ-TREE. The tree was rooted using the Mesorhizobium spp. sequences as the outgroup. The scale bar represents the number of expected substitutions per site under the best-fitting GTR2+FO+R5 model. The same tree, but with collapsed clades, is presented in Figure 2. [file 12864_2022_8662_MOESM3_ESM.pdf]
