# Supplementary material for: Genomic analysis provides novel insights into diversification and taxonomy of Allorhizobium vitis (i.e. Agrobacterium vitis)
Source: BMC Genomics. 2022 Jun 22;23:462. doi: 10.1186/s12864-022-08662-x (PMC9219206; doi:10.1186/s12864-022-08662-x)
Supplement: Supplementary file 4 — Additional file 4: Fig. S4. Heatmap representation of the average nucleotide identity (ANIb) for TAR regions of All. vitis species complex strains. PyANI program Version 0.2.9 (https://github.com/widdowquinn/pyani) was used to calculate ANIb values and generate the clustered heatmap. [file 12864_2022_8662_MOESM4_ESM.pdf]

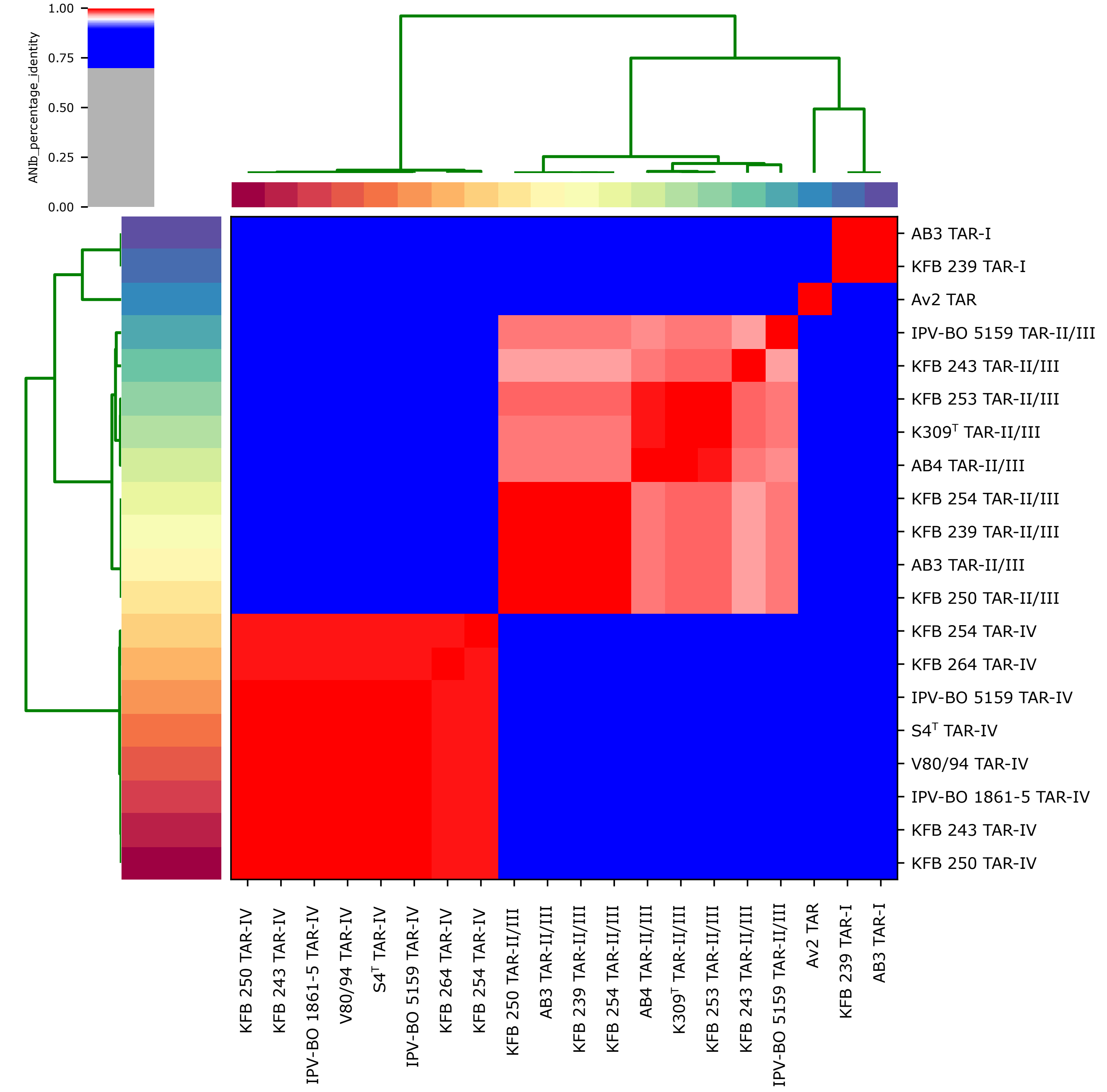

Fig. S4. Heatmap representation of the average nucleotide identity (ANIb) for TAR regions of *All. vitis* species complex strains. PyANI program Version 0.2.9 (https://github.com/widdowquinn/pyani) was used to calculate ANIb values and generate the clustered heatmap.
