# Supplementary material for: Genomic analysis provides novel insights into diversification and taxonomy of Allorhizobium vitis (i.e. Agrobacterium vitis)
Source: BMC Genomics. 2022 Jun 22;23:462. doi: 10.1186/s12864-022-08662-x (PMC9219206; doi:10.1186/s12864-022-08662-x)
Supplement: Supplementary file 5 — Additional file 5: Fig. S5. Score-oriented dendrogram showing the similarity of the MALDI-TOF mass spectra of 14 All. vitis species complex strains studied. The dendrogram was created using the MALDI Biotyper Compass Explorer software (Bruker, Version 4.1.90). [file 12864_2022_8662_MOESM5_ESM.pdf]

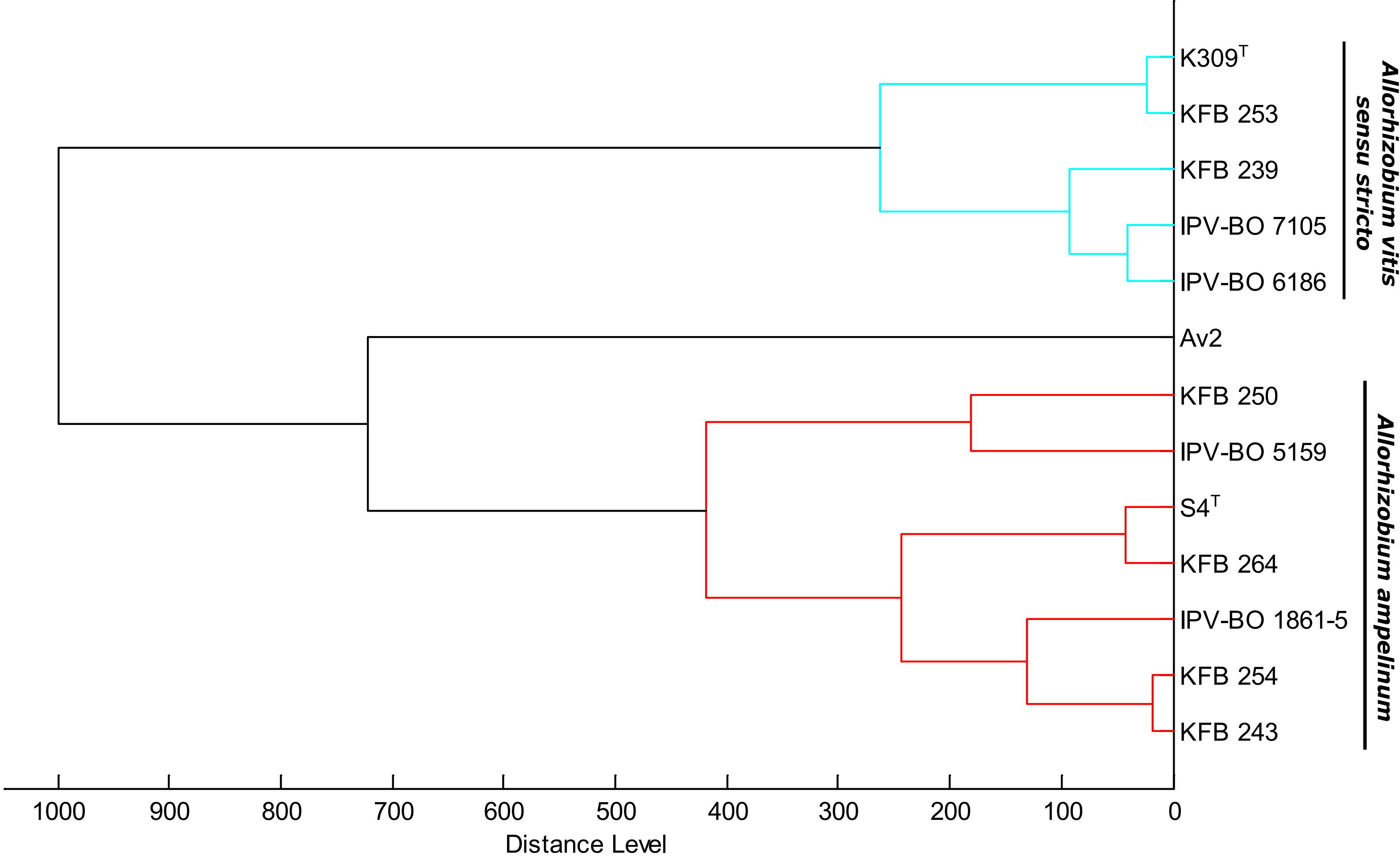

Fig. S5. Score-oriented dendrogram showing the similarity of the MALDI-TOF mass spectra of 14 *All. vitis* species complex strains studied. The dendrogram was created using the MALDI Biotyper Compass Explorer software (Bruker, Version 4.1.90).
